# Fetal calf serum heat inactivation and lipopolysaccharide contamination influence the human T lymphoblast proteome and phosphoproteome

**DOI:** 10.1186/1477-5956-9-71

**Published:** 2011-11-15

**Authors:** Hazir Rahman, Muhammad Qasim, Frank C Schultze, Michael Oellerich, Abdul R Asif

**Affiliations:** 1 Department of Clinical Chemistry, University Medical Centre, Goettingen, Germany; 2 Department of Microbiology, Kohat University of Science and Technology, Kohat, Pakistan

**Keywords:** CCRF-CEM cells, FCS heat inactivation, LPS, proteome, phosphoproteome

## Abstract

**Background:**

The effects of fetal calf serum (FCS) heat inactivation and bacterial lipopolysaccharide (LPS) contamination on cell physiology have been studied, but their effect on the proteome of cultured cells has yet to be described. This study was undertaken to investigate the effects of heat inactivation of FCS and LPS contamination on the human T lymphoblast proteome. Human T lymphoblastic leukaemia (CCRF-CEM) cells were grown in FCS, either non-heated, or heat inactivated, having low (< 1 EU/mL) or regular (< 30 EU/mL) LPS concentrations. Protein lysates were resolved by 2-DE followed by phospho-specific and silver nitrate staining. Differentially regulated spots were identified by nano LC ESI Q-TOF MS/MS analysis.

**Results:**

A total of four proteins (EIF3M, PRS7, PSB4, and SNAPA) were up-regulated when CCRF-CEM cells were grown in media supplemented with heat inactivated FCS (HE) as compared to cells grown in media with non-heated FCS (NHE). Six proteins (TCPD, ACTA, NACA, TCTP, ACTB, and ICLN) displayed a differential phosphorylation pattern between the NHE and HE groups. Compared to the low concentration LPS group, regular levels of LPS resulted in the up-regulation of three proteins (SYBF, QCR1, and SUCB1).

**Conclusion:**

The present study provides new information regarding the effect of FCS heat inactivation and change in FCS-LPS concentration on cellular protein expression, and post-translational modification in human T lymphoblasts. Both heat inactivation and LPS contamination of FCS were shown to modulate the expression and phosphorylation of proteins involved in basic cellular functions, such as protein synthesis, cytoskeleton stability, oxidative stress regulation and apoptosis. Hence, the study emphasizes the need to consider both heat inactivation and LPS contamination of FCS as factors that can influence the T lymphoblast proteome.

## Introduction

Fetal calf serum (FCS) is a complex nutritional supplement that is routinely used in cell culture media [1,2]. Along with the growth factors, FCS contains several complement proteins [3-5]. Proteins of the complement system play a central role in innate immunity [6] and when present in cell culture media, they can influence immunological assays [7,8]. Heat inactivation of serum at 56°C for 30 minutes is used to inhibit the haemolytic activity of serum by decreasing the titer of heat labile complement proteins [9]. There are conflicting reports regarding the significance of FCS heat inactivation before its use in cell culture medium. Several studies have reported that heat inactivation of serum modifies growth factor content and increases cell proliferation [10,11]. However, Leshem and co-workers reported that heat inactivation of serum did not influence lymphocyte functions at least in *in vitro *studies [12].

Bacterial lipopolysaccharide (LPS) is an inevitable contaminant of serum used in cell culture medium. LPS acts via the Toll-like receptor (TLR) complex, which transduces the LPS signal across the plasma membrane and triggers downstream signaling, leading to the secretion of pro-inflammatory cytokines and induction of complement pathways [13-15]. Protein phosphorylation is crucial for gene regulation, cell growth and homeostasis [16,17]. LPS influences proteins by altering their phosphorylation status through activation of various kinases e.g., p70 S6 kinase [18]. The p70 S6 kinase is the downstream effector of the mammalian target of rapamycin complex 1 (mTORC1), an important regulator of cell growth, proliferation, protein synthesis and cell survival [19,20]. Analogous to the effects of FCS heat inactivation, there are contradictory findings regarding the effect of LPS concentrations on the physiology of cultured cells. Some research groups have reported a direct influence of LPS on cellular physiology [21-23], while others have not found any detectable effect on the growth of various cell lines including WI-38, 3T3 and CHO even after using LPS concentrations up to 100 EU/mL [15,24-26]. The heat inactivation procedure itself exerts no deactivating effect on LPS [27].

Cell cultures are routinely used to conduct important biological studies. Often, studies have used varying culture conditions with respect to FCS heat inactivation, or poorly documented LPS concentrations in cell cultures, while not acknowledging their possible effects on the proteome of the cultured cells. The present study was designed to determine the effect of FCS heat inactivation and the concentration of LPS in serum on cultured human T lymphoblastic leukaemia cells employing a proteomic and phosphoproteomic approach.

## Results

Human T lymphoblastic cells were grown in RPMI-1640 medium supplemented either with (a) non-heat inactivated FCS with normal concentrations of LPS (NHE), (b) heat inactivated FCS containing normal concentrations of LPS (HE), (c) non-heat inactivated FCS with low concentrations of LPS (NHL), or (d) heat inactivated FCS with low concentrations of LPS (HL). The cells were grown for at least five passages, harvested and used for 2-DE. The 2-DE gels were silver stained followed by phospho specific staining, and differentially regulated spots were excised, digested, and identified by nano LC Q-TOF MS/MS analysis.

### Cells grown in medium with heat inactivated FCS

**Figure 1:**
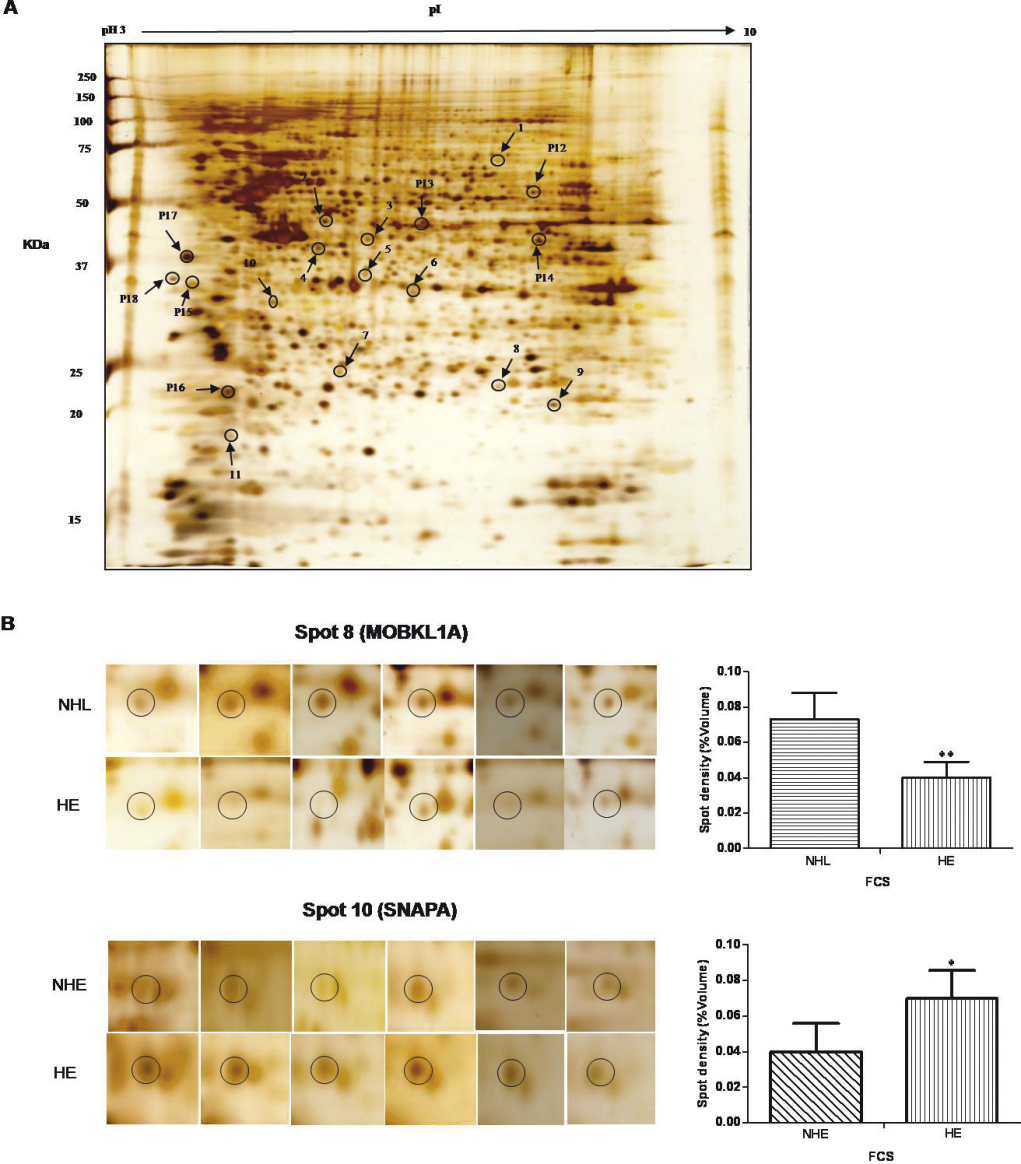
**Silver nitrate stained 2-DE gel**. (A) Proteins (160 μg) were separated in the first dimension using non-linear pH 3-10 gradient IPG strips (17 cm, Bio-Rad), followed by second dimension on 12.5% SDS-PAGE. Consistently regulated spots were excised from silver stained gel after densitometric analysis for identification by Q-TOF MS/MS analysis. Spots marked on the gel showed differentially regulated proteins. Note: "P" refers to phospho protein spots also shown in figure 2. (B) Two representative differentially regulated 2-DE spots (MOBKL1A, spot 8; SNAPA, spot 10) in non-heated FCS with low LPS (NHL) and in heated FCS with normal LPS concentration (HE) respectively. The spot IDs correspond to the listing in Table 1. The error bars represent mean ± SD (*= p < 0.05, **= p < 0.005) of six independent experiments.

**Figure 2:**
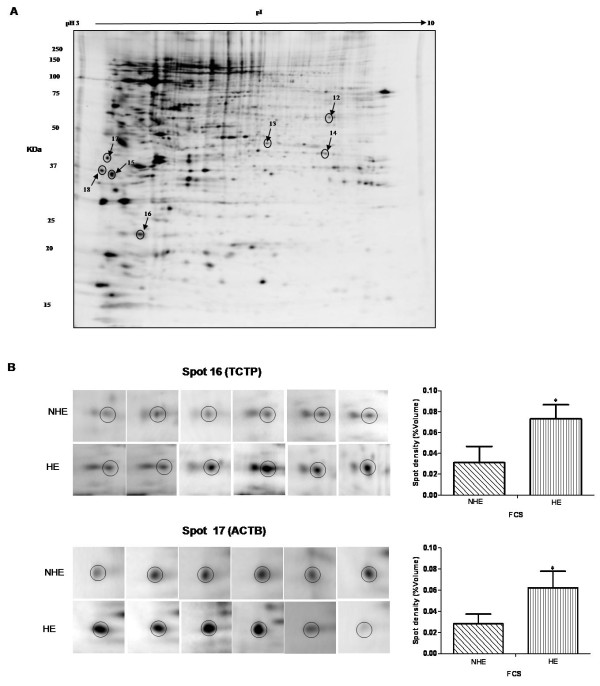
**Phospho-specific florescence stained 2-DE**. (A) Proteins were resolved on 2-DE and gels were stained by Pro-Q Diamond phospho-stain (Invitrogen) and then scanned (FLA -5100). The spots showing significant regulation after densitometry analysis were marked and identified by Q-TOF MS/MS analysis. (B) Illustration of two representative 2-DE spots (TCTP, spot 16; ACTB; spot 17) in non-heated FCS with normal LPS (NHE) and in heated FCS with normal LPS (HE). The error bars represent mean ± SD (*= *p *< 0.05) of six independent experiments.

Initially, we compared human T lymphoblastic cells grown in NHE and HE medium. Four protein spots (numbers 4, 6, 7 and 10 in Table 1 Additional file 1 Figure S1) were up-regulated in the HE group. These were identified as eukaryotic translation initiation factor 3 subunit M (EIF3M), 26S protease regulatory subunit 7 (PRS7), proteasome subunit beta type-4 (PSB4) and alpha-soluble NSF attachment protein (SNAPA) respectively. Figure 1A shows a representative silver stained gel with these differentially regulated spots marked, while Figure 1B shows six replicates of two regulated spots (spots 8 and 10). Densitometric analysis of phospho-stained gels was performed to check the proteins exhibiting significant changes in phosphorylation signals by after heat inactivation of FCS (Figure. 2A). In the HE group, six protein spots 12, 13, 15, 16, 17 and 18 displayed higher phosphorylation signals, identified as T-complex protein 1 subunit delta (TCPD), actin aortic smooth muscle (ACTA), nascent polypeptide-associated complex subunit alpha (NACA), translationally-controlled tumor protein (TCTP), actin cytoplasmic 1 (ACTB) and methylosome subunit pICln (ICLN) respectively (Table 2 Additional file 1 Figure S2). Figure 2 shows a representative phospho-stained gel (Figure 2A) and six replications (Figure 2B) of two differentially phosphorylated proteins (TCTP, spot 16) and (ACTB, spot 17).

**Table 1:** Differentially regulated proteins by LPS and heat inactivation of FCS

Spot ID	**Abb**.	Protein name^a^	**Acc. No**.	Mass (kDa)	Fold change (Mean ± SD)				
					
					HE/NHE	NHL/NHE	HL/NHE	NHL/HE	HL/HE
1	SYFB	PhenylalanyltRNA synthetase beta chain	Q9NSD9	66.1	NS	*2.35 ↓ (0.019 ± 0.01/0.04 ± 0.02)	NS	NS	**2.07 ↓ (0.018 ± 0.007/ 0.037 ± 0.008)
2	QCR1	Cytochrome b-c1 complex subunit 1	P31930	52.6	NS	NS	NS	NS	*1.73 ↓ (0.042 ± 0.012/ 0.074 ± 0.018)
3	SUCB1	Succinyl-CoA ligase subunit beta	Q9P2R7	50.3	NS	NS	NS	NS	*1.50 ↓ (0.55 ± 0.007/ 0.083 ± 0.017)
4	EIF3M	Eukaryotic translation initiation factor 3 subunit M	Q7L2H7	42.5	**1.79 ↑ (0.103 ± 0.013/ 0.057 ± 0.011)	NS	NS	NS	NS
5	NAGK	N-acetyl-D-glucosamine kinase	Q9UJ70	37.3	NS	NS	*1.60 ↑ (0.064 ± 0.024/ 0.04 ± 0.007)	NS	NS
6	PRS7	26S protease regulatory subunit 7	P35998	48.6	*1.69 ↑ (0.104 ± 0.018/ 0.061 ± 0.035)	NS	NS	NS	NS
7	PSB4	Proteasome subunit beta type-4	P28070	29.2	*1.53 ↑ (0.087 ± 0.022/ 0.057 ± 0.017)	NS	NS	NS	NS
8	MOBKL1A	Mps one binder kinase activator A1	Q7L9L4	25.0	NS	NS	NS	**1.85 ↑ (0.076 ± 0.0150/ 0.04 ± 0.009)	NS
9	SOD2	Superoxide dismutase [Mn], mitochondrial	P04179	24.7	NS	NS	NS	*1.68 ↓ (0.08 ± 0.037/ 0.135 ± 0.028)	NS
10	SNAPA	Alpha-soluble NSF attachment protein	P54920	33.2	*1.70 ↑ (0.068 ± 0.015/ 0.04 ± 0.015)	NS	NS	NS	NS
11	DBLOH	Diablo homolog, mitochondrial	Q9NR28	7.1	NS	NS	*2.02 ↑ (0.074 ± 0.008/ 0.033 ± 0.019)	NS	NS

Abb: Abbreviation; Acc. No: Accession number; Mass: Molecular mass of the protein observed in a Mascot search (www.matrixscience.com);

^a ^Proteins identified by Q-TOF MS/MS analysis and database search against Swissprot; ↑: Up-regulated, ↓: Down-regulated, NS: Non-significant change; *= *p *< 0.05, **= *p *< 0.005.

**Table 2:** Differentially phosphorylated proteins by LPS and heat inactivation of FCS

Spot ID	**Abb**.	Protein name ^a^	**Acc. No**.	Mass (kDa)	Fold change (Mean ± SD)				
					
					HE/NHE	NHL/NHE	HL/NHE	NHL/HE	HL/HE
12	TCPD	T-complex protein 1 subunit delta	P50991	57.8	*1.62 ↑ (0.219 ± 0.047/0.135 ± 0.053)	NS	NS	*1.68 ↓ (0.130 ± 0.068/0.219 ± 0.046)	NS
13	ACTA	Actin, aortic smooth muscle	P62736	41.9	*1.83 ↑ (0.108 ± 0.036/0.059 ± 0.029)	NS	NS	NS	NS
14	ADHX	Alcohol dehydrogenase class-3	P11766	39.6	NS	NS	NS	NS	*1.75 ↑ (0.226 ± 0.08/0.129 ± 0.046)
15	NACA	Nascent polypeptide-associated complex subunit alpha	Q13765	23.3	*2.60 ↑ (0.283 ± 0.095/0.108 ± 0.031)	NS	NS	*1.74 ↓ (0.162 ± 0.044/0.283 ± 0.095)	NS
16	TCTP	Translationally-controlled tumor protein	P13693	19.5	*2.30 ↑ (0.073 ± 0.013/0.031 ± 0.015)	NS	NS	NS	NS
17	ACTB	Actin, cytoplasmic 1	Q96HG5	41.7	*2.2 0 ↑ (0.062 ± 0.015/0.028 ± 0.009)	NS	NS	*2.37 ↓ (0.018 ± 0.011/0.043 ± 0.015)	NS
18	ICLN	Methylosome subunit pICln	P54105	26.1	*2.08 ↑ (0.232 ± 0.089/0.111 ± 0.081)	NS	NS	*1.80 ↓ (0.283 ± 0.117/0.510 ± 0.113)	NS

Abb: Abbreviation; Acc. No: Accession number; Mass: Molecular mass of the protein observed in MASCOT search; ^a ^Phospho-proteins identified by Q-TOF MS/MS analysis and database search against Swissprot; ↑: Up-regulated, ↓: Down-regulated, NS: Non-significant change; *= *p *< 0.05, **= *p *< 0.005.

### Proteins with altered expression as a function of FCS-LPS concentrations

We investigated the influence of LPS concentrations on the cell proteome by comparing the NHL with NHE groups, one protein, phenylalanyl tRNA synthetase beta chain (SYFB, spot 1), was down-regulated. In the HE compared to the HL group, three protein spots (spot 1, 2 and 3), identified as SYFB, cytochrome b-c1complex subunit 1-mitochondrial (QCR1) and succinyl-CoA ligase subunit beta-mitochondrial (SUCB1), were up-regulated (Table 1 Additional file 1 Figure S1). In phospho-stained gels only one protein, alcohol dehydrogenase class-3 (ADHX, spot 14), was down-regulated in the HE compared to the HL group (Table 2).

### Proteins regulated by both LPS concentration and heat treatment of FCS

**Figure 3:**
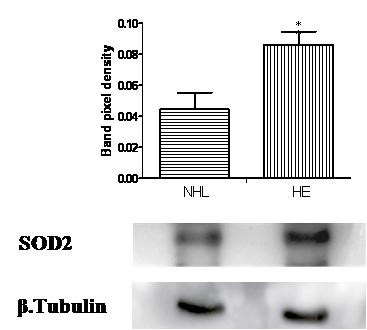
**Immunoblot analysis of superoxide dismutase 2 (SOD2) expression**. CCRF-CEM lysate treated with non-heated FCS with low LPS (NHL) and in heated FCS with normal LPS (HE), were resolved on 1DE and immunoblotted with antibody against SOD2. Densitometric analyses were done using Lab Image version 2.71 software. β-tubulin was used as a loading control. The error bars represent mean ± SD (*= *p *< 0.05) of three independent experiments.

The HL group compared to the NHE demonstrated two up-regulated proteins (spot 5 and 11) identified as N-acetyl-D-glucosamine kinase (NAGK) and Diablo homolog mitochondrial (DBLOH). By comparing the NHL and HE groups, one protein (spot 8) Mps one binder kinase activator-like 1A (MOBKL1A) was up-regulated (Figure 1B), whereas another protein (spot 9) identified as superoxide dismutase 2 (SOD2) was down-regulated. Regulation of SOD2 expression was further confirmed by immunoblot analysis (Figure 3). The MS/MS spectra for all differentially regulated proteins in silver and phospho-stained gels are provided as "Additional file 2 Table S1, Table S2".

## Discussion

Cell culture media are supplemented with FCS as a source of growth factors necessary for cell survival and cell proliferation [2,28,29]. Besides growth factors, FCS also contains complement proteins and growth inhibitory factors [3,30]. Heat inactivation of FCS is considered a mandatory step in cell culture to inactivate serum inhibitory factors [9,31]; however, such heat treatment has no effect on the activity of LPS [27]. Recently E. Manor reported an enhancement of cell proliferation by human plasma as compared to human serum [32]; however others prefer the use of serum to supplement cell culture medium [33,34]. There are at least 18 different factors including 11 chemokines which are reported to be more abundant in serum as compared to plasma; these are likely to be released by platelets during the coagulation cascade [35]. FCS is believed to be more effective in stimulating cell proliferation than human serum (HS) or rabbit serum [10,36]. Depending on the cancerous cell type, the LPS may have varied effects on cell physiology [22,23,37-39]. The present study used a proteomics approach to investigate whether heat treatment and LPS concentration exert any detectable changes on the global proteome expression and phosphoproteome in cultured human T cells. It is important to stress that we examined both heat inactivated and non-heat inactivated FCS each with regular and low LPS concentrations. Most commercially available FCS has less than 30 EU/mL of LPS. To mimic practices commonly used in the laboratories, we used regular and low LPS containing FCS.

### Impact of heat inactivation of FCS on protein regulation

In the present study, four proteins displayed increased levels in the heat inactivated LPS group, as compared to non-treated group (Table 1). EIF3M (spot 4), an important regulator of protein turnover [40] was up-regulated. This finding correlates with a previous study investigating the influence of serum heat inactivation on cell protein content in osteoblast progenitor cells [10]. However, LPS concentration (NHL compared to NHE) had no significant influence on EIF3M expression. Two proteins (PRS7 "spot 6" and PSB4 "spot 7"), which are members of a multiprotein complex involved in cellular protein degradation [41] were up-regulated by heat inactivation of FCS. The expression of both proteins remained unchanged at both low and normal LPS concentrations in FCS. This is in line with the previous observation that at least ≥ 100 ng/mL of LPS was required to influence the expression of PRS7 [42]. FCS heat inactivation influenced the intensity of the phosphorylation signal of six proteins (TCTP, TCPD, NACA, ACTA, ACTB and ICLN). TCTP (spot 16) is a cytoskeletal related protein involved in cell growth, survival and protection against various stress conditions [43]. Cells grown in heat inactivated FCS supplemented medium showed increased TCTP phosphorylation as compared to non-heat inactivated group (Figure 2B). The phosphorylation of TCTP is linked to a decrease in microtubule stabilization and could potentially affect microtubule dynamics, resulting in compromised structural integrity of cells [44]. TCPD protein showed increased phosphorylation in the heat inactivated FCS group. TCPD is a member of the chaperone containing T-complex polypeptide 1 (CCT) that is involved in both protein folding and cytoskeleton network regulation [45]. This protein also helps in dopamine mediated neuronal apoptosis [46]. Another protein, NACA (spot 15) was up-regulated in the heat inactivated FCS group. NACA is a transcriptional co-activator that modulates c-Jun-mediated transcription [47]. Two cytoskeletal proteins ACTA and ACTB (spot 13 and 17 respectively) displayed increase phosphorylation signals in the HE group, as compared to NHE group. These proteins are ubiquitously expressed in eukaryotic cells, are involved in the cytoskeletal architecture of the cell [48], and they are modified by phosphorylation during stressful conditions [49]. The ICLN protein (spot 18) participates in the regulation of small nuclear ribonucleoproteins, (snRNP) biogenesis, and is an essential component of spliceosomes [50]. It showed an altered phosphorylation signal in the presence of FCS heat inactivation.

### Impact of LPS contamination in FCS on protein regulation

Three proteins, SYFB (spot 1 involved in protein biosynthesis [51]). QCR1 (spot 2, a mitochondrial respiratory chain protein [52] and SUCB1 (spot 3, which is the mitochondrial matrix enzyme involved in the ATP synthesis [53] were significantly up-regulated when grown in medium containing normal (ie "regular") as compared to low LPS concentrations. This implies that increased LPS concentrations may have stimulatory effects on protein synthesis. These findings are consistent with observations made by Hamilton and colleagues, who reported increased protein synthesis in murine peritoneal macrophages cultured at 10 ng/ml LPS concentration [54]. LPS has been reported to induce protein synthesis in B lymphocytes [55,56], and enhance T lymphocyte proliferation [57] by an unknown molecular mechanism.

### Protein regulation by combined changes in LPS concentrations and heat treatment of FCS

Two proteins, NAGK (spot 5, which converts N-acetylglucosamine into GlcNAc 6-phosphate [58]) and DBLOH (spot 11, which is a pro-apoptotic protein [59]) were up-regulated in the HL as compared to the NHE group. Cells grown in medium containing non-heated FCS with low LPS had significantly increased expression of MOBKL1A (spot 8, a cell division associated protein [60]) (Figure 1B). SOD2 (spot 9 is a mitochondrial anti-oxidant enzyme essential for cell survival [61]) that protects T lymphocytes against free oxygen radicals that are generated in these cells to kill microorganisms [62]. In the NHL group SOD2 expression was down-regulated as compared to HE, both in the 2-DE and immunoblot analysis (Figure 3). This suggests that commonly used (ie. "regular") LPS concentrations and serum heat inactivation might produce oxidative challenge to the cells. Previous reports have also described a similar modulation in the SOD2 expression by LPS in human monocytes [63]. Such cellular proteome regulation reflects a survival strategy of the cells allowing them to respond to external factors through alterations in metabolic activity.

## Conclusion

These results suggest that the heat inactivation and LPS concentrations in FCS are indeed able to alter the expression and phosphorylation of proteins involved in important cellular functions of cultured human T lymphocytes. The study emphasizes the importance of considering the effects of FCS heat treatment, or LPS concentrations used in the cell cultures, on phosphorylation and cellular proteome of T cells. This work also demonstrates the ability of a proteomic approach to demonstrate the complex picture of cellular responses to selected cell culture conditions. The exact mechanism(s) by which serum heat inactivation and LPS regulate cellular protein expression and post-translational modification is not yet clear and needs further investigation.

## Methods

### Reagents

RPMI-1640, FCS containing LPS concentrations of either < 1 EU/mL (< 0.1 to 0.2 ng/mL) or < 30 EU/mL (< 3 to 6 ng/mL), Dulbecco's phosphate buffer saline (PBS), penicillin and streptomycin were obtained from PAA Laboratories, Colbe, Germany. Urea, thiourea, dithiothreitol (DTT), trypsin, triflouroacetic acid (TFA), acitonitrile (ACN) and ammonium bicarbonate (AMBIC) were from Sigma-Aldrich, Steinheim, Germany. CHAPS buffer was from AppliChem, Darmstadt, Germany, and ampholeytes, protein assay reagents, Immobilized pH gradient strips (IPG strips) were provided by Bio-Rad, Munich, Germany. Protease and phosphatase inhibitor cocktail were from Roche, Mannheim, Germany. Bromophenol blue and Tris base were from Carl Roth, Karlsruhe, Germany, and sodium dodecyl sulfate (SDS) was from Serva, Heidelberg, Germany. Glycerin, potassium ferricynaide and sodium thiosulfate were from Merck, Darmstadt, Germany and formic acid from BASF, Ludwigshafen, Germany. Superoxide dismutase 2 (SOD2) antibodies was a gift from Dr. Dihazi, UMG, Goettingen, Germany. β-tubulin antibody was from BioVendor, Heidelberg, Germany and antibodies to HRP labelled anti-mouse secondary antibodies were from Bio-Rad, Munich, Germany.

### Cell cultures

Human T lymphoblastic leukaemia cells (CCRF-CEM) were purchased from DSMZ (German collection of microorganisms and cell cultures, Braunschweig, Germany). Cells were grown in 75 cm^2 ^culture flasks (Sarstedt, Numberecht, Germany) in RPMI-1640 medium containing L-glutamine, 10% FCS, 100,000 U/L penicillin and 100 μg/L streptomycin, in 95% humidity and 5% CO_2 _conditions at 37°C.

### Heat inactivation and LPS treatment of cultured cells

FCS was heated at 56°C for 30 minutes before adding it to the RPMI-1640 medium. CCRF-CEM cells were grown in RPMI-1640 medium supplemented either with (a) FCS without heat inactivation and a normal concentration of LPS (NHE), (b) FCS with heat inactivation containing a normal concentration of LPS (HE), (c) FCS without heat inactivation having a low concentration of LPS (NHL), or (d) heated FCS with low concentration of LPS (HL). The cells were adapted in RPMI-1640 medium supplemented with four different FCS concentrations for at least five passages before starting the first harvest. The cells were grown to a density of 0.25 × 10^6 ^cells/mL under recommended conditions i.e., 37°C, 95% humidity, 20% O_2_, 5% CO_2 _and the medium was changed every second day. All experiments were repeated six times.

### Cell lysis and protein estimation

Cells were washed with ice cold PBS and lysed in lysis buffer (7 M urea, 2 M thiourea, 4% CHAPS, 2% ampholytes [pH 3-10], 1% DTT, 1% protease inhibitor and 1% phosphatase inhibitor cocktail). Protein concentration was measured as described by Bradford (1976) using serum albumin as a standard [64].

### Sample preparation and two-dimensional gel electrophoresis (2-DE)

2-DE was performed as described by Gorg et al [65]. Briefly, a 160 μg protein sample was diluted in rehydration buffer (7 M urea, 2 M thiourea, 4% CHAPS, 0.2 ampholytes [pH 3-10], 0.2% DTT and 0.25% bromophenol blue) were applied on immobilized pH gradient strip (IPG strip, 17 cm) with a non-linear pH range of 3-10 at room temperature overnight for passive rehydration. Isoelectric focusing was performed with a Bio-Rad Protean electrophoresis apparatus set to final 32000 Volts hour. The IPG strip was then equilibrated for 20 minutes in equilibration buffer (6 M urea, 30% glycerol, 2% SDS, and 50 mM Tris-HCl [pH 8.8]) containing DTT (10 g/L) and then subsequently immersed for 20 minutes in fresh equilibration buffer containing iodoacetamide (40 g/L). Following equilibration, proteins were separated by SDS-PAGE at a constant voltage of 100 Volts using a 12.5% polyacrylamide separation gel at 4°C [66].

### Phospho-specific staining of 2-DE gels

The gels were fixed twice in solution containing 50% methanol and 10% acetic acid for 45 minutes and washed three times in double distilled water for 15 minutes each. Gels were incubated in Pro-Q Diamond phospho-stain (Invitrogen, Paisley, UK) overnight in the dark at room temperature, destained three times for 30 minutes in 20% ACN and 50 mM sodium acetate, followed by three washes in double distilled water for five minutes each. Gels were scanned using an imaging instrument (FLA -5100 Fuji photo film, Dusseldorf, Germany) at a wavelength of 532 nm.

### Visualization of proteins and densitometric analysis

Proteins were visualized by silver staining, as described by Blum et al [67], immersed in a fixative solution (50% methanol and 12% acetic acid) for one hour and washed in 50% and 30% ethanol for 20 minutes each. Gels were sensitized in 0.02% sodium thiosulfate for 60 seconds and washed three times in water. Staining was done in silver solution (0.2% silver nitrate, 0.026% formaldehyde) for 20 minutes, followed by three washings in water. All gels were developed in a solution containing 6% sodium carbonate, 0.0185% formaldehyde and 6% sodium thiosulfate until spots appeared and the reaction was stopped by adding the stop solution (50% methanol and 12% acetic acid). Gels were scanned (CanoScan 8400F, Canon, Krefeld, Germany) dried (Gel Drier, Bio-Rad, Munich, Germany), and subjected to densitometric analysis using the Delta2D software version 4.0 (DECODON, Greifswald, Germany).

### Tryptic digestion

Differentially expressed spots were excised and in-gel digested according to the method described by Shevchenko and colleagues [68]. Briefly, sliced gel spots were destained with 30 mM potassium ferricyanide and 100 mM sodium thiosulfate; followed by washing with 50% ACN and 100 mM AMBIC, which was then removed and dried in a vacuum centrifuge (UNIVAPO, uniEquip, Matinsried, Germany). The gel pieces were digested with trypsin digestion buffer (0.1 μg/μl trypsin, 1 M calcium chloride, and 1 M AMBIC) for 45 minutes on ice and then incubated overnight in digestion buffer without trypsin at 37°C. The peptides were extracted with increasing concentrations of ACN and TFA in several rounds and the extracted peptides were dried by vacuum centrifugation. Peptides were reconstituted in 0.1% FA for injection into a nano-flow HPLC.

### Peptide sequence analysis using nano LC ESI Q-TOF MS/M and database search

Peptide samples (1 μl) were introduced onto two consecutive C18-reversed phase chromatography columns (C18 pepMap: 300 μm × 5 mm; 5 μm particle size, and C18 pepMap100 nanoanalytical column: 75 μm × 15 cm; 3 μm particle size; LC Packings, Germering, Germany) using a nano-flow CapLC autosampler (Waters, Eschborn, Germany). Peptides were eluted with an increasing gradient of ACN and analyzed on a Q-TOF Ultima Global mass spectrometer (Micromass, Manchester, UK) equipped with a nanoflow ESI Z-spray source in the positive ion mode, as previously described [69]. The data were analyzed with the MassLynx (version 4.0) software. The peaklists were searched using the online MASCOT search engine (http://www.matrixscience.com) against the UniProt/SwissProt database release 15.15 (515203 entries, 181334896 elements). The data were searched against the database with following parameters: trypsin as enzyme for digestion; up to a maximum of one missed cleavage site allowed; monoisotopic mass value and with unrestricted protein mass; peptide tolerance ± 0.5Da and MS/MS tolerance ± 0.5Da. Proteins were identified on the basis of two or more peptides, whose ions score exceeded the threshold, *p *< 0.05 which reflects the 95% confidence level for the matched peptides.

### SDS-PAGE and Western blotting

Samples were resolved on 12.5% SDS-PAGE and electro-transferred using a semi-dry transblot system (SD transblot, Bio-Rad, Munich, Germany) onto PVDF membrane (Millipore, Schwalbach, Germany) at 17 Volts in a transfer buffer (192 mM glycine, 10% methanol, and 25 mM Tris [pH 8.3]) for 30 minutes. The membrane was blocked with 5% skimmed milk powder prepared in TBS-T buffer (50 mM Tris-HCl [pH 7.5], 200 mM NaCl, and 0.05% Tween 20) for one hour at room temperature and washed three times with TBS-T buffer. Membrane was incubated with Anti-SOD2, or anti-β tubulin antibody prepared in 5% skimmed milk powder for overnight at 4°C. After three washes in TBS-T for five minutes each, the membrane was incubated in HRP labelled anti-mouse secondary antibody for one hour at room temperature. Followed by subsequent washes, the signal on the blot was detected using an enhanced chemiluminescent (ECL) reagent (GE Healthcare, Munich, Germany) and developed on Amersham Hyperfilm (GE Healthcare, Munich, Germany). Signal intensities from each immunoblot were quantified using Lab Image software version 2.71 (Kapelan, Leipzig, Germany).

### Statistical analysis

Densitometric analysis of protein spots from silver and phospho-stained gel were performed using Delta2D software. Protein spots, which showed ≥ 1.5 fold change in phosphorylation signal and consistently statistically significant (*p *< 0.05) using the Student's *t*-test in at least six independent 2-DE experiments, were selected for in-gel digestion and identified using ESI Q-TOF MS/MS analysis. Error bars in results represent mean ± SD. Immunoblot intensities were quantified using LabImage software (Kapelan, Leipzig, Germany). Immunoblotting was repeated at least three times and results were expressed as mean ± SD with significance measured using the Student's *t*-test (*p *< 0.05).

## Competing interests

The authors declare that they have no competing interests.

## Authors' contributions

HR carried out the experiments and drafted the manuscript. MQ contributed to the data analysis. FCS helped to draft the manuscript. ARA and MO participated in the design, supervision and interpretation of the study. All authors read and approved the final manuscript.

## Supplementary Material

Additional file 1 **Figure S1. Graphical display of selected proteins significantly regulated in CCRF-CEM cells in silver stained 2-DE gel**. CCRF-CEM cell lysates were resolved on 2-DE and gels were stained with silver nitrate. Significantly regulated protein spots by densitometric analysis were identified my Q-TOF MS/MS analysis. (A) Bar graphs represent mean spot density for four proteins which were up-regulated in HE (heat inactivation with regular LPS) group as compared to NHE (No heat inactivation with regular LPS) control group. (B) Three proteins were up-regulated in HE (heat inactivation with regular LPS) as compared to HL (heat inactivation with low LPS) group. Bar charts illustrate mean spot density. The error bars represent ± SD (*= *p *< 0.05, **= *p *< 0.005) of six independent experiments. **Figure S2. Proteins significantly regulated in CCRF-CEM cells in phospho-specific stained 2-DE gel**. CCRF-CEM cell lysates were separated on 2-DE and gels were stained with phospho-specific stain. Differentially regulated protein spots by densitometric analysis were identified by Q-TOF MS/MS analysis. Bar graphs show mean spot density for four proteins which were up-regulated in HE (heat inactivation with regular LPS) group as compared to NHE (No heat inactivation with regular LPS) control group. The error bars represent ± SD (*= *p *< 0.05) of six independent experiments. Click here for file

Additional file 2 **Table S1. MS/MS spectral data of differentially regulated proteins identified by Q-TOF analysis**. Accession number, score and MS/MS spectra of identified proteins. **Table S2. MS/MS spectral data of differentially regulated phospho-proteins identified by Q-TOF analysis**. Accession number, score and MS/MS spectra of identified proteins. Click here for file
