# Supplementary material for: Fetal calf serum heat inactivation and lipopolysaccharide contamination influence the human T lymphoblast proteome and phosphoproteome
Source: Proteome Sci. 2011 Nov 15;9:71. doi: 10.1186/1477-5956-9-71 (PMC3280938; doi:10.1186/1477-5956-9-71)

**Additional file 1, Figure S1.**

**Graphical display of selected proteins significantly regulated in CCRF-CEM cells in silver stained 2-DE gel.**


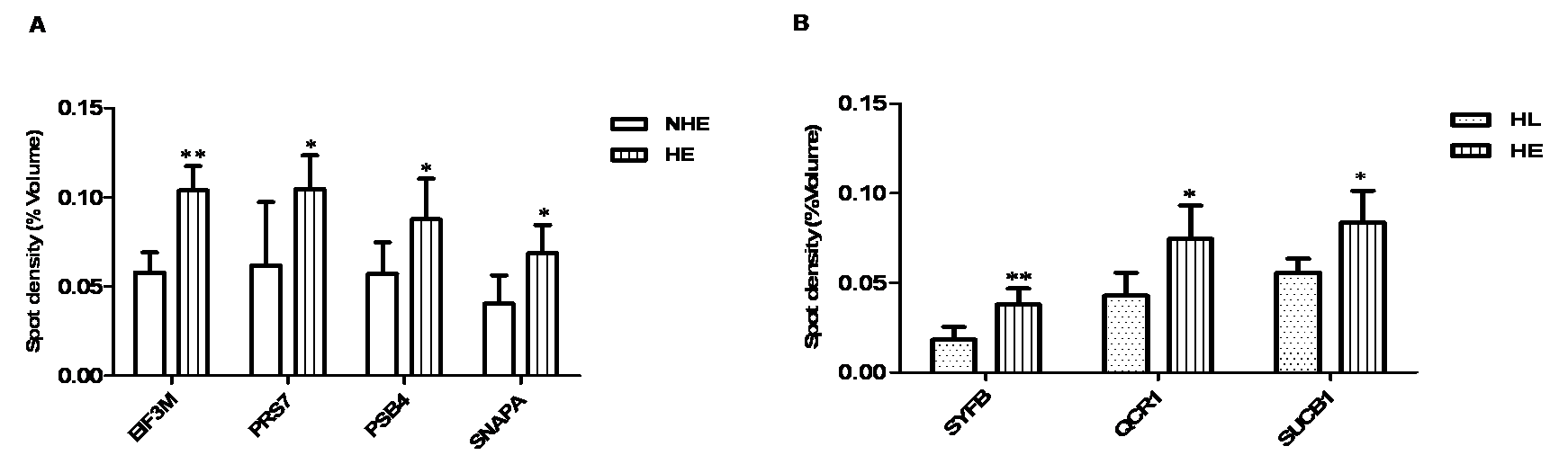


**Additional file 1, Figure S2.**

**Phospho-proteins significantly regulated in CCRF-CEM cells in phospho-specific stained 2-DE gel.**


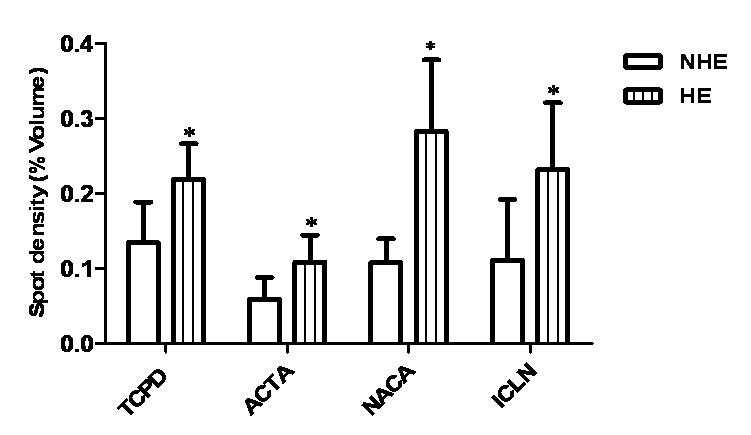

Supplement: Additional file 1 — Figure S1. Graphical display of selected proteins significantly regulated in CCRF-CEM cells in silver stained 2-DE gel. CCRF-CEM cell lysates were resolved on 2-DE and gels were stained with silver nitrate. Significantly regulated protein spots by densitometric analysis were identified my Q-TOF MS/MS analysis. (A) Bar graphs represent mean spot density for four proteins which were up-regulated in HE (heat inactivation with regular LPS) group as compared to NHE (No heat inactivation with regular LPS) control group. (B) Three proteins were up-regulated in HE (heat inactivation with regular LPS) as compared to HL (heat inactivation with low LPS) group. Bar charts illustrate mean spot density. The error bars represent ± SD (*= p < 0.05, **= p < 0.005) of six independent experiments. Figure S2. Proteins significantly regulated in CCRF-CEM cells in phospho-specific stained 2-DE gel. CCRF-CEM cell lysates were separated on 2-DE and gels were stained with phospho-specific stain. Differentially regulated protein spots by densitometric analysis were identified by Q-TOF MS/MS analysis. Bar graphs show mean spot density for four proteins which were up-regulated in HE (heat inactivation with regular LPS) group as compared to NHE (No heat inactivation with regular LPS) control group. The error bars represent ± SD (*= p < 0.05) of six independent experiments. [file 1477-5956-9-71-S1.DOC]
