# Supplementary material for: Fetal calf serum heat inactivation and lipopolysaccharide contamination influence the human T lymphoblast proteome and phosphoproteome
Source: Proteome Sci. 2011 Nov 15;9:71. doi: 10.1186/1477-5956-9-71 (PMC3280938; doi:10.1186/1477-5956-9-71)
Supplement: Additional file 2 — Table S1. MS/MS spectral data of differentially regulated proteins identified by Q-TOF analysis. Accession number, score and MS/MS spectra of identified proteins. Table S2. MS/MS spectral data of differentially regulated phospho-proteins identified by Q-TOF analysis. Accession number, score and MS/MS spectra of identified proteins. [file 1477-5956-9-71-S2.DOC]

**Additional file 2, Table S1.**

**MS/MS analysis table for differentially regulated proteins identified by Q-TOF MS/MS analysis**

**Footnotes:** ^a^Mascot score a = >42 indicate identification or extensive homology (p < 0.05).

^b^Peptide matched b= Number of peptides matched with protein in MS/MS query.

| **Spot ID** | **Protein** | **Accession no.** | **Mascot**  **score^a^** | **Peptide^b^** | **MS/MS Analysis** |
| --- | --- | --- | --- | --- | --- |
| 1 | SYFB | Q9NSD9 | 195 | 7 | **1** MPTVSVKR**DL LFQALGR**TYT DEEFDELCFE FGLELDEITS EKEIISKE  **51** NVKAAGASDV VLYKIDVPAN RYDLLCLEGL VRGLQVFKER **IKAPVYK**RVM  **101** PDGKIQK**LII TEETAK**IRPF AVAAVLRNIK FTKDRYDSFI ELQEKLHQNI  **151** CRKRALVAIG THDLDTLSGP FTYTAKRPSD IKFKPLNKTK EYTACELMNI  **201** YKTDNHLKHY LHIIENKPLY PVIYDSNGVV LSMPPIINGD HSRITVNTRN  **251** IFIECTGTDF TKAKIVLDII VTMFSEYCEN QFTVEAAEVV FPNGKSHTFP  **301** ELAYRKEMVR ADLINKKVGI RETPENLAKL LTRMYLKSEV IGDGNQIEIE  **351** IPPTRADIIH ACDIVEDAAI AYGYNNIQMT LPKTYTIANQ FPLNKLTELL  **401** RHDMAAAGFT EALTFALCSQ EDIADKLGVD ISATKAVHIS NPK**TAEFQVA**  **451 R**TTLLPGLLK TIAANRKMPL PLK**LFEISDI VIK**DSNTDVG AKNYRHLCAV  **501** YYNKNPGFEI IHGLLDR**IMQ LLDVPPGEDK** GGYVIK**ASEG PAFFPGR**CAE  **551** IFARGQSVGK LGVLHPDVIT KFELTMPCSS LEINIGPFL  MS/MS Fragmentation of **ASEG PAFFPGR**  **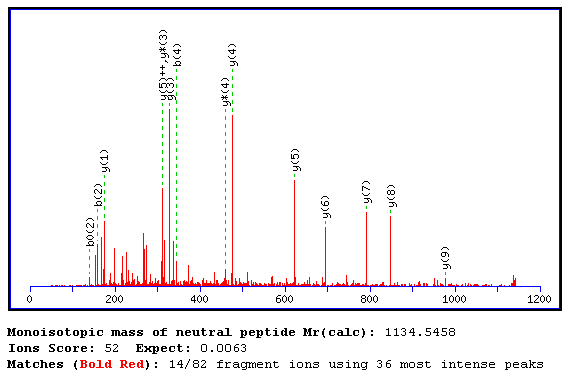** |
| 2 | QCR1 | P31930 | 190 | 14 | **1** MAASVVCRAA TAGAQVLLRA RRSPALLRTP ALRSTATFAQ ALQFVPETQV  **51** SLLDNGLRVA SEQSSQPTCT VGVWIDVGSR FETEKNNGAG YFLEHLAFKG  **101** TK**NRPGSALE KEVESMGAHL NAYSTREHTA YYIK**ALSKDL PKAVELLGDI  **151** VQNCSLEDSQ IEKERDVILR **EMQENDASMR** DVVFNYLHAT AFQGTPLAQA  **201** VEGPSENVRK LSR**ADLTEYL STHYK**APRMV LAAAGGVEHQ QLLDLAQKHL  **251** GGIPWTYAED AVPTLTPCR**F TGSEIR**HRDD ALPFAHVAIA VEGPGWASPD  **301** NVALQVANAI IGHYDCTYGG GVHLSSPLAS GAVANKLCQS FQTFSICYAE  **351** TGLLGAHFVC DRMKIDDMMF VLQGQWMR**LC TSATESEVAR** GKNILR**NALV**  **401 SHLDGTTPVC EDIGRSLLTY GRRIPLAEWE SRIAEVDASV VR**EICSKYIY  **451** DQCPAVAGYG PIEQLPDYNR IRSGMFWLRF  MS/MS Fragmentation of **LCTSATESEVAR**  **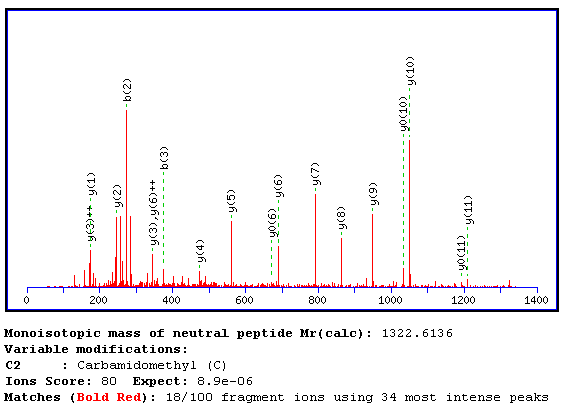** |
| 3 | SUCB1 | Q9P2R7 | 192 | 8 | **1** MAASMFYGRL VAVATLRNHR PRTAQRAAAQ VLGSSGLFNN HGLQVQQQQQ  **51** RNLSLHEYMS MELLQEAGVS VPKGYVAK**SP DEAYAIAK**KL GSKDVVIKAQ  **101** VLAGGRGKGT FESGLKGGVK **IVFSPEEAK**A VSSQMIGKKL FTKQTGEKGR  **151** ICNQVLVCER KYPRREYYFA ITMERSFQGP VLIGSSHGGV NIEDVAAESP  **201** EAIIKEPIDI EEGIKK**EQAL QLAQK**MGFPP NIVESAAENM VKLYSLFLKY  **251** DATMIEINPM VEDSDGAVLC MDAK**INFDSN SAYR**QKKIFD LQDWTQEDER  **301** DKDAAKANLN YIGLDGNIGC LVNGAGLAMA TMDIIKLHGG TPANFLDVGG  **351** GATVHQVTEA FKLITSDKKV LAILVNIFGG IMRCDVIAQG IVMAVKDLEI  **401** KIPVVVRLQG TRVDDAK**ALI ADSGLK**ILAC DDLDEAARMV VK**LSEIVTLA**  **451 K**QAHVDVKFQ LPI  MS/MS Fragmentation of **SPDEAYAIAK**  **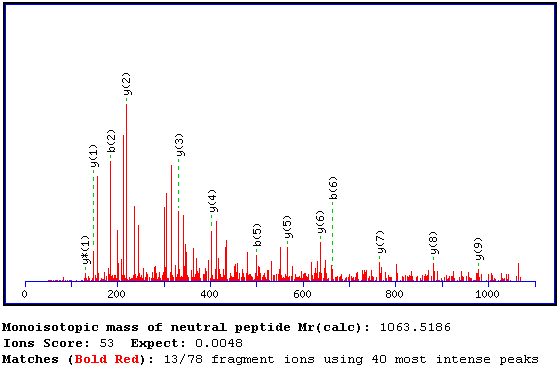** |
| 4 | EIF3M | Q7L2H7 | 89 | 3 | **1** MSVPAFIDIS EEDQAAELRA YLKSKGAEIS EENSEGGLHV DLAQIIEACD  **51** VCLKEDDKDV ESVMNSVVSL LLILEPDKQE ALIESLCEKL VK**FREGERPS**  **101 LR**LQLLSNLF HGMDKNTPVR YTVYCSLIKV AASCGAIQYI PTELDQVRKW  **151** ISDWNLTTEK KHTLLRLLYE ALVDCKKSDA ASKVMVELLG SYTEDNASQA  **201** RVDAHRCIVR ALKDPNAFLF DHLLTLKPVK FLEGELIHDL LTIFVSAKLA  **251** SYVKFYQNNK DFIDSLGLLH EQNMAKMR**LL TFMGMAVENK** EISFDTMQQE  **301** LQIGADDVEA FVIDAVRTKM VYCKIDQTQR KVVVSHSTHR TFGKQQWQQL  **351** YDTLNAWKQN LNKVKNSLLS LSDT  MS/MS Fragmentation of **LLTFMGMAVENK**  **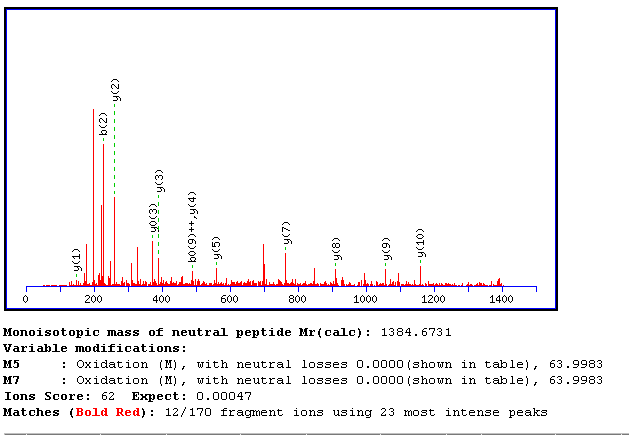** |
| 5 | NAGK | Q9UJ70 | 58 | 3 | **1** MAAIYGGVEG GGTR**SEVLLV SEDGK**ILAEA DGLSTNHWLI GTDKCVER**IN**  **51 EMVNR**AKRKA GVDPLVPLRS LGLSLSGGDQ EDAGR**ILIEE LR**DRFPYLSE  **101** SYLITTDAAG SIATATPDGG VVLISGTGSN CRLINPDGSE SGCGGWGHMM  **151** GDEGSAYWIA HQAVKIVFDS IDNLEAAPHD IGYVKQAMFH YFQVPDRLGI  **201** LTHLYRDFDK CRFAGFCRKI AEGAQQGDPL SRYIFRKAGE MLGRHIVAVL  **251** PEIDPVLFQG KIGLPILCVG SVWKSWELLK EGFLLALTQG REIQAQNFFS  **301** SFTLMKLRHS SALGGASLGA RHIGHLLPMD YSANAIAFYS YTFS  MS/MS Fragmentation of **SEVLLVSEDGK**  **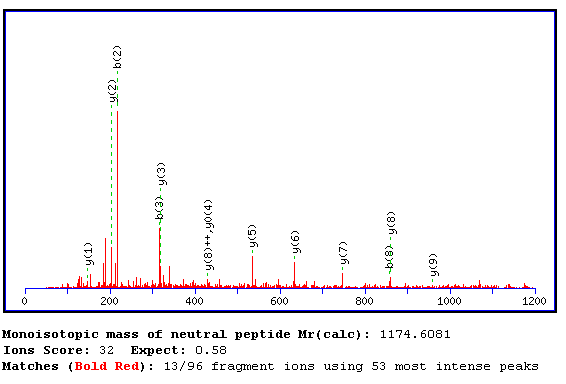** |
| 6 | PRS7 | P35998 | 115 | 4 | **1** MPDYLGADQR KTKEDEKDDK PIRALDEGDI ALLK**TYGQST YSR**QIKQVED  **51** DIQQLLKKIN ELTGIKESDT GLAPPALWDL AADKQTLQSE QPLQVARCTK  **101** IINADSEDPK YIINVKQFAK FVVDLSDQVA PTDIEEGMRV GVDRNKYQIH  **151** IPLPPKIDPT VTMMQVEEKP DVTYSDVGGC KEQIEKLREV VETPLLHPER  **201** FVNLGIEPPK GVLLFGPPGT GKTLCARAVA NRTDACFIRV IGSELVQKYV  **251** GEGARMVREL FEMARTKKAC LIFFDEIDAI GGAR**FDDGAG GDNEVQRTML**  **301 ELINQLDGFD PR**GNIKVLMA TNRPDTLDPA LMRPGRLDRK IEFSLPDLEG  **351** RTHIFKIHAR SMSVERDIRF ELLARLCPNS TGAEIRSVCT EAGMFAIRAR  **401** RKIATEKDFL EAVNKVIKSY AKFSATPRYM TYN  MS/MS Fragmentation of **FDDGAGGDNEVQR**  **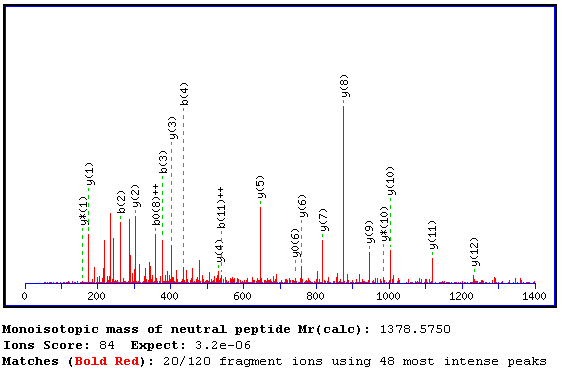** |
| 7 | PSB4 | P28070 | 180 | 5 | **1** MEAFLGSRSG LWAGGPAPGQ FYRIPSTPDS FMDPASALYR GPITR**TQNPM**  **51 VTGTSVLGVK** FEGGVVIAAD MLGSYGSLAR FRNISRIMRV NNSTMLGASG  **101** DYADFQYLKQ VLGQMVIDEE LLGDGHSYSP RAIHSWLTRA MYSRRSKMNP  **151** LWNTMVIGGY ADGESFLGYV DMLGVAYEAP SLATGYGAYL AQPLLR**EVLE**  **201 KQPVLSQTEA R**DLVERCMRV LYYRDARSYN R**FQIATVTEK** GVEIEGPLST  **251** ETNWDIAHMI SGFE  MS/MS Fragmentation of **TQNPMVTGTSVLGVK** **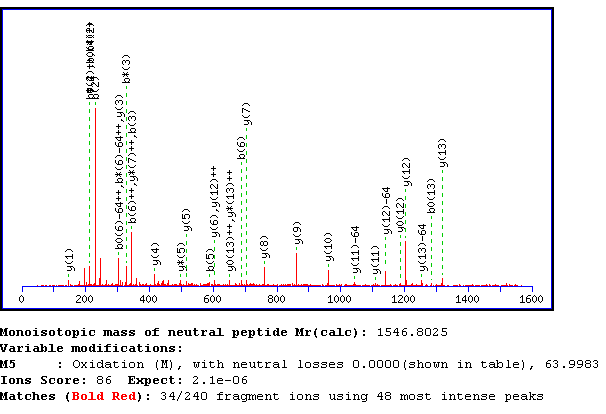** |
| 8 | MOBKL1A | Q7L9L4 | 118 | 2 | **1** MSFLFGSRSS KTFKPKKNIP EGSHQYELLK **HAEATLGSGN LR**MAVMLPEG  **51** EDLNEWVAVN TVDFFNQINM LYGTITDFCT EESCPVMSAG PKYEYHWADG  **101** TNIKKPIKCS APKYIDYLMT WVQDQLDDET LFPSKIGVPF PKNFMSVAKT  **151** ILKRLFRVYA HIYHQHFDPV IQLQEEAHLN TSFKHFIFFV QEFNLIDRR**E**  **201 LAPLQELIEK** LTSKDR  MS/MS Fragmentation of **HAEATLGSGNLR**  **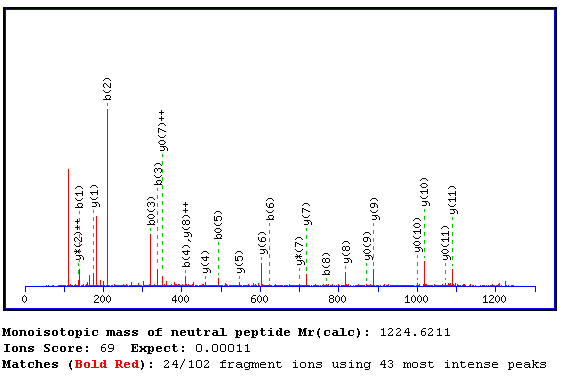** |
| 9 | SOD2 | P04179 | 126 | 4 | **1** MLSRAVCGTS RQLAPALGYL GSRQKHSLPD LPYDYGALEP HINAQIMQLH  **51** HSKHHAAYVN NLNVTEEKYQ EALAK**GDVTA QIALQPALK**F NGGGHINHSI  **101** FWTNLSPNGG GEPK**GELLEA IKR**DFGSFDK FKEKLTAASV GVQGSGWGWL  **151** GFNKERGHLQ IAACPNQDPL QGTTGLIPLL GIDVWEHAYY LQYK**NVRPDY**  **201 LK**AIWNVINW ENVTERYMAC KK  MS/MS Fragmentation of **GDVTAQIALQPALK**  **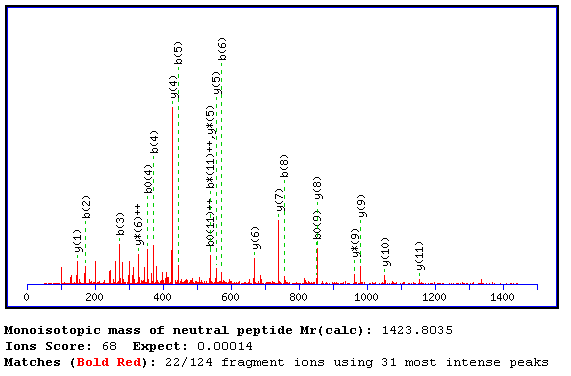** |
| 10 | SNAPA | P54920 | 44 | 3 | **1** MDNSGKEAEA MALLAEAERK VK**NSQSFFSG LFGGSSK**IEE ACEIYARAAN  **51** MFKMAKNWSA AGNAFCQAAQ LHLQLQSKHD AATCFVDAGN AFKKADPQEA  **101** INCLMRAIEI YTDMGRFTIA AKHHISIAEI YETELVDIEK AIAHYEQSAD  **151** YYKGEESNSS ANKCLLKVAG YAALLEQYQK AIDIYEQVGT NAMDSPLLKY  **201** SAKDYFFKAA LCHFCIDMLN AKLAVQKYEE LFPAFSDSRE CKLMKKLLEA  **251** HEEQNVDSYT ESVK**EYDSIS R**LDQWLTTML LRIKK**TIQGD EEDLR**  MS/MS Fragmentation of **TIQGDEEDLR**  **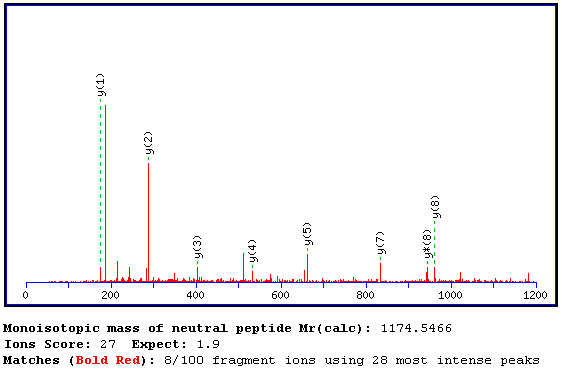** |
| 11 | DBLOH | Q9NR28 | 52 | 2 | **1** MAALKSWLSR SVTSFFRYRQ CLCVPVVANF KKRCFSELIR PWHKTVTIGF  **51** GVTLCAVPIA QKSEPHSLSS EALMRRAVSL VTDSTSTFLS QTTYALIEAI  **101** TEYTKAVYTL TSLYRQYTSL LGKMNSEEED EVWQVIIGAR AEMTSKHQEY  **151** LKLETTWMTA VGLSEMAAEA AYQTGADQAS ITAR**NHIQLV K**LQVEEVHQL  **201** SRKAETK**LAE AQIEELR**QKT QEEGEERAES EQEAYLRED  MS/MS Fragmentation of **LAEAQIEELR**  **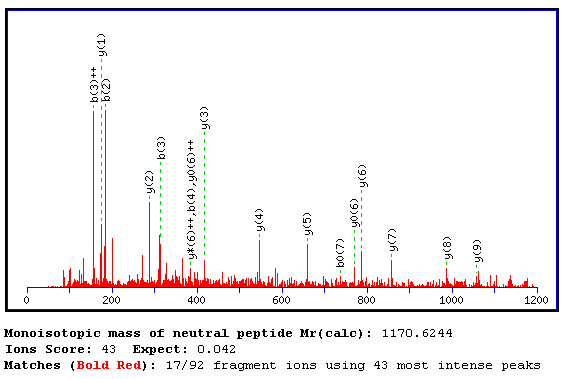** |

**Additional file 2, Table S2.**

**MS/MS analysis table for differentially regulated phospho-proteins identified by Q-TOF MS/MS analysis**

**Footnotes:** ^a^Mascot score a = >42 indicate identification or extensive homology (p < 0.05).

^b^Peptide matched b= Number of peptides matched with protein in MS/MS query.

| **Spot ID** | **Protein** | **Accession no.** | **Mascot**  **score^a^** | **Peptides^b^** | **MS/MS Analysis** |
| --- | --- | --- | --- | --- | --- |
| 12 | TCPD | P50991 | 152 | 6 | **1** MPENVAPRSG ATAGAAGGRG KGAYQDRDKP AQIRFSNISA AKAVADAIRT  **51** SLGPKGMDKM IQDGKGDVTI TNDGATILKQ MQVLHPAAR**M LVELSK**AQDI  **101** EAGDGTTSVV IIAGSLLDSC TKLLQKGIHP TIISESFQKA LEKGIEILTD  **151** MSRPVELSDR ETLLNSATTS LNSKVVSQYS SLLSPMSVNA VMK**VIDPATA**  **201 TSVDLR**DIKI VKKLGGTIDD CELVEGLVLT QKVSNSGITR VEKAKIGLIQ  **251** FCLSAPKTDM DNQIVVSDYA QMDRVLREER AYILNLVKQI KKTGCNVLLI  **301** QKSILR**DALS DLALHFLNK**M KIMVIKDIER EDIEFICKTI GTKPVAHIDQ  **351** FTADMLGSAE LAEEVNLNGS GKLLKITGCA SPGKTVTIVV RGSNK**LVIEE**  **401 AER**SIHDALC VIRCLVKKR**A LIAGGGAPEI ELALR**LTEYS RTLSGMESYC  **451** VRAFADAMEV IPSTLAENAG LNPISTVTEL RNRHAQGEKT AGINVRKGGI  **501** SNILEELVVQ PLLVSVSALT LATETVRSIL K**IDDVVNTR**  MS/MS Fragmentation of **ALIAGGGAPEIELALR**  **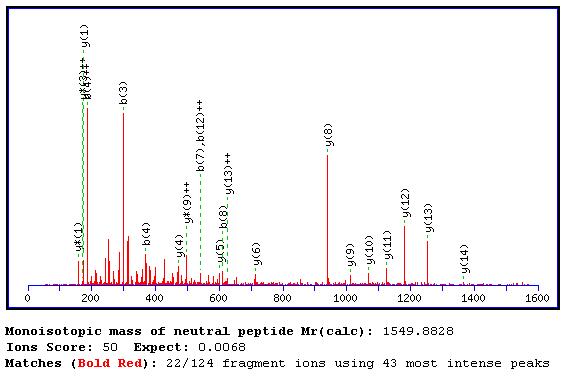** |
| 13 | ACTA | P62736 | 128 | 5 | **1** MCEEEDSTAL VCDNGSGLCK **AGFAGDDAPR AVFPSIVGRP RHQGVMVGMG**  **51 QK**DSYVGDEA QSKRGILTLK YPIEHGIITN WDDMEKIWHH SFYNELRVAP  **101** EEHPTLLTEA PLNPKANREK MTQIMFETFN VPAMYVAIQA VLSLYASGRT  **151** TGIVLDSGDG VTHNVPIYEG YALPHAIMRL DLAGRDLTDY LMKILTERGY  **201** SFVTTAEREI VRDIKEKLCY VALDFENEMA TAASSSSLEK SYELPDGQVI  **251** TIGNERFRCP ETLFQPSFIG MESAGIHETT YNSIMKCDID IRKDLYANNV  **301** LSGGTTMYPG IADRMQK**EIT ALAPSTMK**IK IIAPPERKYS VWIGGSILAS  **351** LSTFQQMWIS KQEYDEAGPS IVHRKCF  MS/MS Fragmentation of **AGFAGDDAPR**  **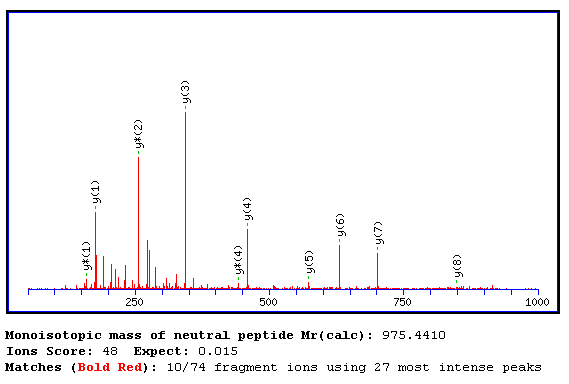** |
| 14 | ADHX | P11766 | 74 | 4 | **1** MANEVIKCKA AVAWEAGKPL SIEEIEVAPP KAHEVRIKII ATAVCHTDAY  **51** TLSGADPEGC FPVILGHEGA GIVESVGEGV TKLKAGDTVI PLYIPQCGEC  **101** KFCLNPKTNL CQKIRVTQGK **GLMPDGTSR**F TCKGKTILHY MGTSTFSEYT  **151** VVADISVAKI DPLAPLDKVC LLGCGISTGY GAAVNTAKLE PGSVCAVFGL  **201** GGVGLAVIMG CKVAGASR**II GVDINKDK**FA RAKEFGATEC INPQDFSKPI  **251** QEVLIEMTDG GVDYSFECIG NVKVMRAALE ACHKGWGVSV VVGVAASGEE  **301** IATRPFQLVT GRTWK**GTAFG GWK**SVESVPK **LVSEYMSK**KI KVDEFVTHNL  **351** SFDEINKAFE LMHSGKSIRT VVKI  MS/MS Fragmentation of **IIGVDINKDK**  **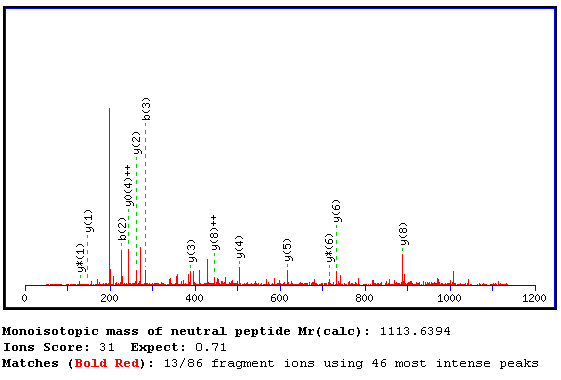** |
| 15 | NACA | Q13765 | 342 | 4 | **1** MPGEATETVP ATEQELPQPQ AETGSGTESD SDESVPELEE QDSTQATTQQ  **51** AQLAAAAEID EEPVSKAKQS RSEKKARKAM SKLGLRQVTG VTRVTIRKSK  **101** **NILFVITKPD VYKSPASDTY IVFGEAKIED LSQQAQLAAA EK**FKVQGEAV  **151** SNIQENTQTP TVQEESEEEE VDETGVEVK**D IELVMSQANV SR**AKAVRALK  **201** NNSNDIVNAI MELTM  MS/MS Fragmentation of **SPASDTYIVFGEAK**  **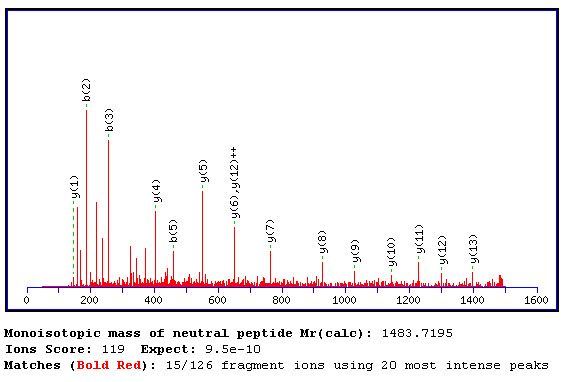** |
| 16 | TCTP | P13693 | 103 | 5 | **1** MIIYRDLISH DEMFSDIYKI REIADGLCLE VEGKMVSRTE GNIDDSLIGG  **51** NASAEGPEGE GTESTVITGV DIVMNHHLQE TSFTKEAYKK **YIKDYMK**SIK  **101** **GKLEEQRPER VKPFMTGAAE QIK**HILANFK NYQFFIGENM NPDGMVALLD  **151** YR**EDGVTPYM IFFK**DGLEME KC  MS/MS Fragmentation of **GKLEEQRPER**  **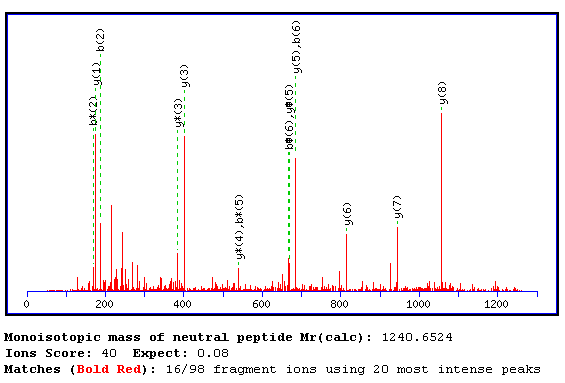** |
| 17 | ACTB | Q96HG5 | 76 | 5 | **1** MDDDIAALVV DNGSGMCK**AG FAGDDAPRAV FPSIVGRPR**H QGVMVGMGQK  **51** DSYVGDEAQS KRGILTLKYP IEHGIVTNWD DMEKIWHHTF YNELRVAPEE  **101** HPVLLTEAPL NPKANREKMT QIMFETFNTP AMYVAIQAVL SLYASGRTTG  **151** IVMDSGDGVT HTVPIYEGYA LPHAILRLDL AGR**DLTDYLM K**ILTERGYSF  **201** TTTAEREIVR DIKEKLCYVA LDFEQEMATA ASSSSLEKSY ELPDGQVITI  **251** GNERFRCPEA LFQPSFLGME SCGIHETTFN SIMKCDVDIR KDLYANTVLS  **301** GGTTMYPGIA DRMQK**EITAL APSTMK**IKII APPERKYSVW IGGSILASLS  **351** TFQQMWISK**Q EYDESGPSIV HR**KCF  MS/MS Fragmentation of **AGFAGDDAPR**  **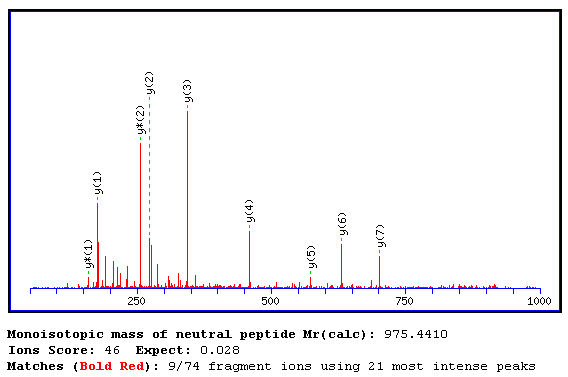** |
| 18 | ICLN | P54105 | 139 | 4 | **1** MSFLK**SFPPP GPAEGLLRQQ PDTEAVLNGK GLGTGTLYIA ESR**LSWLDGS  **51** GLGFSLEYPT ISLHALSRDR SDCLGEHLYV MVNAKFEEES KEPVADEEEE  **101** DSDDDVEPIT EFRFVPSDKS ALEAMFTAMC ECQALHPDPE DEDSDDYDGE  **151** EYDVEAHEQG QGDIPTFYTY EEGLSHLTAE GQATLERLEG MLSQSVSSQY  **201** NMAGVRTEDS IRDYEDGMEV DTTPTVAGQF EDADVDH  MS/MS Fragmentation of **GLGTGTLYIAESR**  **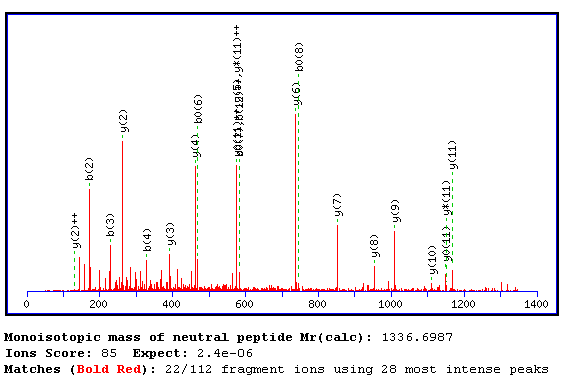** |
